# The Psychosexual Profile of Sexual Assistants: An Internet-Based Explorative Study

**DOI:** 10.1371/journal.pone.0098413

**Published:** 2014-06-11

**Authors:** Erika Limoncin, Debora Galli, Giacomo Ciocca, Giovanni Luca Gravina, Eleonora Carosa, Daniele Mollaioli, Andrea Lenzi, Emmanuele A. Jannini

**Affiliations:** 1 Department of Clinical and Applied Sciences and Biotechnologies, School of Sexology, University of L’Aquila, L’Aquila, Italy; 2 Department of Endocrinology, Sapienza University of Rome, Rome, Italy; 3 Department of Systems Medicine, University of Rome Tor Vergata, Rome, Italy; University of Vienna, Austria

## Abstract

**Introduction:**

Sexual assistance may have some aspects that resemble prostitution and others that might lead one to think of sexual assistants as similar to a group of subjects whose sexual object is disability (devotees). In this study, we investigate whether a rigorous selection and training process on the part of specialised organisations may reduce the risk of training subjects with an atypical sexual interest and behaviours resembling prostitution.

**Materials and Methods:**

The study population consisted of 152 subjects defining themselves as sexual assistants. Subjects were initially contacted on websites specifically dedicated to sexual assistants and prostitutes. One hundred and twenty subjects were selected, by propensity score analysis, and studied by means of a modified version of a semi-structured questionnaire previously developed to investigate a population of subjects attracted by disability.

**Results:**

The study group was composed of 80 trained and 40 untrained sexual assistants, with mean ages of 41.5 (SD +/−12.58) and 44.5 (SD +/−11.62), respectively. A significant number of untrained subjects affirmed that their motivation in carrying out sexual assistance was of a remunerative nature, while this number was lower among the trained assistants (p = 0.001). Nearly all untrained subjects claimed to do one or more of the following activities during sexual assistance: sexual intercourse, oral sex, and masturbation. Among the trained subjects, however, only 47.5% claimed to do one or more of these activities, which means that there is a significant gap between trained and untrained assistants (p<0.0001). The existence of an atypical sexual interest was more evident between untrained rather than between trained subjects (p<0.0001).

**Conclusions:**

Sexual assistance represents a way through which people affected by disabilities may attain the right to explore their sexuality in a safe setting. This can be guaranteed only if sexual assistants are trained and carefully selected by specialised organisations.

## Introduction

The lives of persons affected by disabilities, and especially the lives of those affected by intellectual disabilities, are often characterised by the lack of a job, meaningful activities, and close relationships [1], [2]. The sexuality of disabled people is affected even more profoundly.

Social attitudes toward the sexuality of people affected by disabilities are changing, shifting the general opinion from a concept of handicapped people as asexual individuals or as potential sex offenders, to a concept of disabled people having natural sexual needs and rights [2]. However, the contemporary social model of disability still pays attention to sexually-related civil rights, such as the right to marriage, procreation, and protection against unconditional sterilisation, rather than to the way in which people affected by disabilities can satisfy their sexual drives [3]. Despite the shift in opinion, the acceptance of a physical sexuality associated with individuals affected by an intellectual disability remains difficult. In fact, masturbation, which is often the only way for disabled individuals to satisfy their own sexual needs, is generally associated with the stereotypical idea of pathological masturbation performed in public places [4].

On the basis of this background, it is easy to understand how people affected by disabilities live their sexuality, which is often also compromised by limited opportunities to meet other people and by the difficulty of negotiating possible relationships. In this social context, sexual assistants may represent an option to satisfy sexual needs and to give these subjects, who otherwise may have little chance of being sexually active, the opportunity to improve their sexual health.

The sexual assistant is in most cases a professional figure. These assistants are trained by specialised organisations after a rigorous selection process which includes psychological and mental health assessments. Hence, the sexual assistant is learned about disability and the modes of connecting with people affected by disabilities, aiming at their sexual needs through caresses and massages and ultimately masturbatory activities. However, people affected by disabilities wishing sexual intercourse require the help of local prostitutes, who are for the most part untrained with respect to the special needs of men and women with disabilities.

Sexual assistants work in many European countries, such as Denmark, Germany, Netherlands, and Spain, where they have a proper legal status. In other countries, such as France, sexual assistants do not yet have a proper legal status, while in Switzerland sexual assistants have a status similar to that of prostitutes. Regardless of how sexual assistance is legally defined, many believe that it bears a close similarity to prostitution since this service is paid for and the costs are met entirely by the “client,” with the main difference being in the area of expertise. However, although untrained prostitutes may receive clients with disabilities, sexual assistants are more highly regarded by parents of people affected by disabilities, who believe that people affected by disabilities need more time and contact than able-bodied people.

Sexual assistants are more similar to a group of individuals who have an atypical sexual attraction for disability. Recently, we described a population with a fetishistic attraction to disability. Although sexual assistants, carefully selected by associations and professionally trained, teach disabled people to experience their sexuality in a safeguarded setting, it is possible that untrained subjects providing sexual services may belong to this fetishistic population and represent “predators” for people with disabilities.

Thus, the entire field of sexual assistance is still largely unknown and a matter of debate.

## Aims

The primary aim of this study was to verify whether specific aspects of prostitution were equally present in groups of professionally trained and untrained subjects who claimed to work as sexual assistants.

The secondary aims were (i) to investigate whether sexual assistants had characteristics suggestive of an atypical sexual interest, and (ii) to determine if this sexual interest was more recurring in the group of subjects professionally trained or in the group spontaneously undertaking this job.

## Materials and Methods

### Subjects

This internet-based survey was developed in the period between June 2011 and March 2012. Given the covert nature of the study population, it was difficult to select a representative sample of sexual assistants. These difficulties are mainly related to the evidence that (i) the population of sexual assistants is virtually unknown and variously estimated; (ii) sexual assistance may consist of different activities; (iii) the population is likely to include professionally trained as well as opportunistic or untrained workers who may provide their services spontaneously for a fee or gift but may not be explicitly involved with commercial or professional sexual assistance; and (iv) while sexual assistants in commercial premises are likely to be accessible, opportunistic or untrained sexual assistants are more difficult to contact.

Thus, our study population was selected via a cross-sectional convenience sample (non probability sampling) using snowballing as the main method of recruitment, as suggested for studies with study populations like the one analysed here [5].

The study population was composed of participants (males and females) who claimed to be 18 years of age or over and defined themselves as sexual assistants. Another inclusion criterion observed while searching for the sample was the ability to speak English, German, or Italian.

Subjects were initially contacted on websites specifically dedicated to sexual assistants and prostitutes (Appendix S1). All known sources were used to individuate sexual assistants, and recruitment strategies included collaboration with key individuals and associations involved in the training of sexual assistants. Individuals who initially agreed to participate in the survey were then contacted by personal email. Participants received a detailed description of the study aims and design and of the level of commitment to which they would be agreeing. The participants were unpaid and willingly took part in the research. This study was performed in accordance with the Declaration of Helsinki and the European Union Guidelines on Good Clinical Practice, and was approved by the local ethical committee. Written informed consent was obtained from each participant.

### Criteria for the Definition of Sexual Assistance, Prostitution, Intellectual Disability, and Atypical Sexual Interest

The sexual assistant was defined as any person who provides sexual assistance to physically and/or intellectually disabled people. By sexual assistance we mean one or more of these activities: sexual intercourse, oral sex, massage therapy including erotic massage, masturbatory acts, and discussion of sexuality, contraception, and the appropriate use of sex toys.

The sex worker (prostitute) was defined as any person who engages in the provision to another person, under an arrangement of a commercial or remunerative nature, of any of the following sexual activities: sexual intercourse, oral sex, masturbation, and any activity, other than the aforementioned, which involves physical contact and the use of one person by another for her/his sexual satisfaction [6], [7].

Intellectual disability can be considered as a condition for which the intelligence level is below-average and the skills necessary for day-to-day living are inadequate.

Sexual assistance can be confused with prostitution only when its remunerative or commercial character is coupled with one of the following activities: oral sex, masturbatory activities, or any activity, other than the aforementioned, which involves physical contact and the use of one person by another for her/his sexual satisfaction.

A subject with an atypical sexual interest was defined as any person who had a recurrent sexual fantasy or behaviour involving the object of her/his own sexual desire (the person affected by a disability) irrespective of the presence of distress.

### The Semi-structured Questionnaire and the Kinsey Scale

A semi-structured questionnaire in English, German, and Italian was developed at the University of L’Aquila. Part of this semi-structured questionnaire was previously used to study a population of subjects who are attracted by disability and called devotees [8]. In the present study, additional aspects were investigated in order to verify the existence of specific elements characterising sex work and atypical sexual interest in a population composed of trained and untrained sexual assistants.

The following questions were used to investigate the activities carried out during sexual assistance and their commercial or remunerative nature: “What are your motivations to work as a sexual assistant?” with the answer choices being 1) for money, 2) to turn my sexual interest into a job, 3) to take care of people affected by disabilities, and 4) I don’t know; “Is your work as a sexual assistant always remunerated by money or other kinds of gifts?” with 1) Yes, 2) No, and 3) I don’t know as the answer choices; and “What kind of activities do you carry out during sexual assistance?” which was an open question.

Among the different characteristics which indicate an atypical sexual interest [9], recurring sexual fantasies, behaviours involving the object of the sexual assistant’s own sexual desire, and the presence of distress were chosen for investigation.

The presence of recurring fantasies and distress were investigated by the following questions: “Do you experience recurrent sexual fantasies about or sexual attraction to disabled people, or sexual pleasure during sexual assistance for people affected by disabilities?” with 1) Yes, 2) No, and 3) I don’t know as the answer choices and “Do you feel personal distress about your sexual attraction to people affected by disabilities?” with 1) Yes, 2) No, and 3) I don’t know as the answer choices.

In addition, the reasons why disability is found attractive were investigated by the following open question: “What do you find most attractive about people with disabilities?”.

Finally, we used the Kinsey Scale [10], a largely used measure in the attempt to describe sexual orientation, with scores ranging from 0 (exclusively heterosexual) to X (nonsexual).

### Statistical Methods

Continuous variables were condensed by means and standard deviation (SD). Differences in continuous variables were analysed by the Student’s t-test when two groups were compared. Binary variables were condensed as absolute or relative frequencies. Differences in dichotomous variables were analysed with the Chi-squared test or Fisher’s exact test when appropriate. In order to reduce treatment selection bias and determine treatment effects, a matched propensity analysis was performed. Multivariate logistic regression was used to calculate the predicted probability of the dependent variables as well as the propensity score for each observation in the data set. In particular, the use of propensity analysis in observational, nonrandomized studies, permits to balance the groups in order to reduce the effects of confounding. The dependent variables included in the multivariate analysis are listed in Table 1. A 1∶2 matched analysis was performed in which one untrained subject was matched to two trained subjects. For the matched analysis, differences between matched pairs were evaluated using a t-test for paired data for continuous variables and the McNemar’s test for binary data. P values<0.05 were considered statistically significant. The SPSS version 13.0 software package was used for all statistical analysis and graphic presentations.

**Table 1:** The table shows the clinical variables between groups according to propensity score matching.

VARIABLES	TRAINED SUBJECTS (N = 80)	UNTRAINED SUBJECTS (N = 40)	P value
**Age (mean; SD)**	**41.7 (2.58)**	**44.5 (11.62)**	**0.244**
**Provenance (%; N)**			
Europe	**38.7%; (31/80)**	**40%; (16/40)**	**0.947**
North/Central America	**31.2%; (25/80)**	**30%; (12/40)**	**0.944**
Oceania	**18.7%; (15/80)**	**22.5%; (9/40)**	**0.809**
Africa	**7.5%; (6/80)**	**7.5%; (3/40)**	**0.840**
I’d rather not say	**3.7%; (3/80)**	**0%; (0/40)**	**0.535**
**Educational level (%; N)**			
Junior High School	**10%; (8/80)**	**10%; (4/40)**	**0.747**
Senior High School	**48.7%; (39/80)**	**47.5%; (19/40)**	**0.949**
University degree	**37.5%; (30/80)**	**37.5%; (15/40)**	**0.841**
I’d rather not say	**3.7%; (3/80)**	**17.5%; (2/40)**	**0.872**
**Kinsey Scale (%; N)** *			
Score 0	**38.7%; (31/80)**	**45%; (18/40)**	**0.646**
Score 1	**18.7%; (15/80)**	**22.5%; (9/40)**	**0.809**
Score 2	**20%; (16/80)**	**10%; (4/40)**	**0.260**
Score 3	**13.7%; (11/80)**	**10%; (4/40)**	**0.770**
Score 4	**2.5%; (2/80)**	**10%; (4/40)**	**0.183**
Score 5	**1.2%; (1/80)**	**0%; (0/40)**	**0.723**
Score 6	**5%; (4/80)**	**2.5%; (1/40)**	**0.872**
Score X	**0%; (0/80)**	**0%; (0/40)**	**1**

*Kinsey Scale: Score 0: “Exclusively heterosexual”; Score 1: “Predominantly heterosexual, only incidentally homosexual”; Score 2: “Predominantly heterosexual, but more than incidentally homosexual”; Score 3: “Equally heterosexual and homosexual”; Score 4: “Predominantly homosexual, but more than incidentally heterosexual”; Score 5: “Predominantly homosexual, only incidentally heterosexual”; Score 6: “Exclusively homosexual”; Score X: “Nonsexual”.

## Results

**Figure 1:**
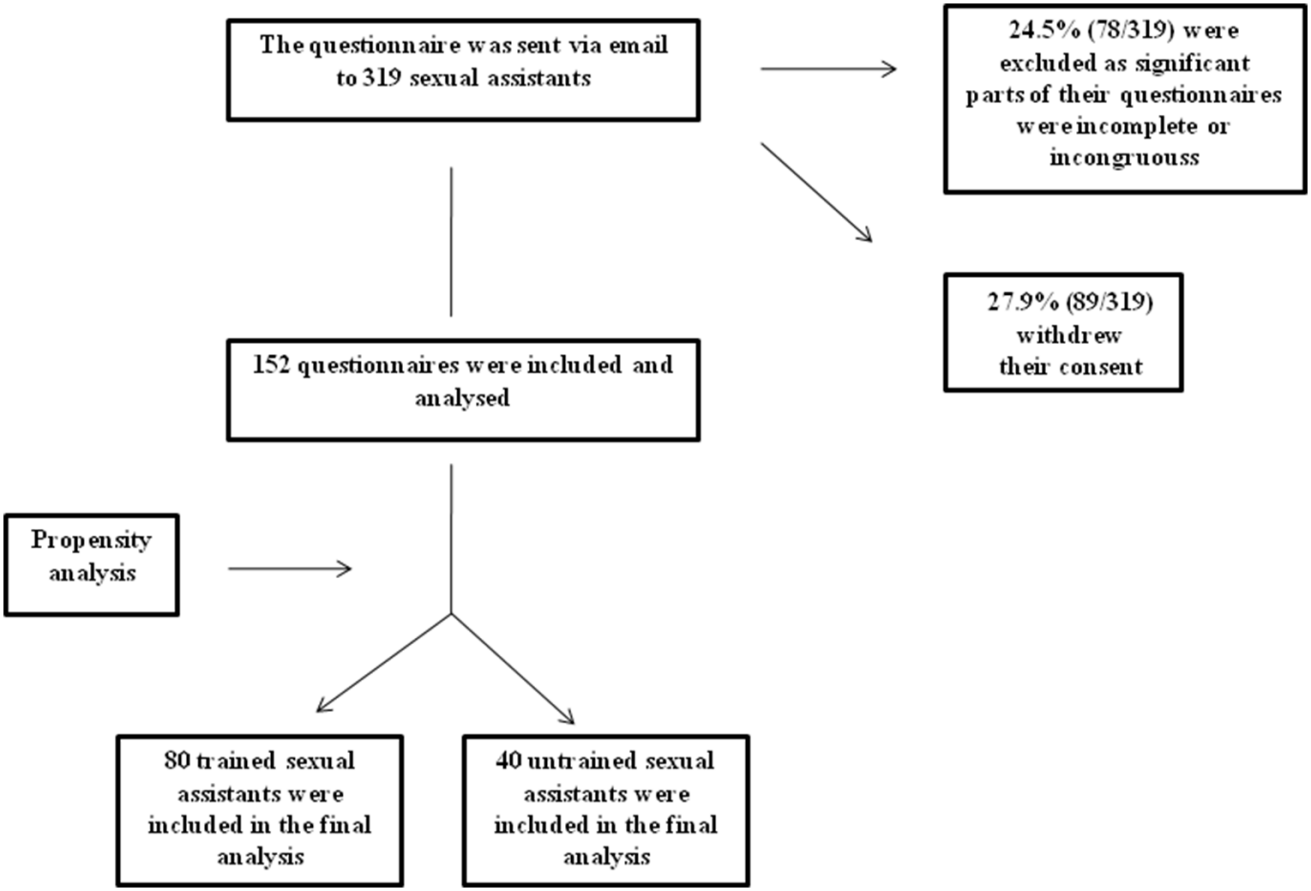
In this figure is represented the sexual assistants’ acceptance rate.

The sexual assistants’ acceptance rate is represented in Figure 1.

More than half the participants (63.1%; 96/152) were recruited from associations organising educational courses for sexual assistance. All subjects belonging to this group were considered professionally trained. The remaining subjects (36.8%; 56/152) were recruited from associations of prostitutes, or individually through word of mouth. This last group did not receive professional training to practice sexual assistance.

About half the sexual assistants (59.2%; 90/152) reported working with people affected both by physical and intellectual disability.

For the comparative analysis between trained and untrained sexual assistants regarding the presence of characteristics suggestive of prostitution and atypical sexual interest, a propensity score analysis was performed. The population analysed consisted of 152 subjects and their completed questionnaires. After propensity analysis selection, the number of subjects included in the comparative analysis consisted of 120 participants and their questionnaires. This number was achieved by excluding 32 subjects who did not present overlapping propensity score values.

The final sample of sexual assistants was composed of 80 trained and 40 untrained sexual assistants with mean ages of 41.5 (SD +/−12.58) and 44.5 (SD +/−11.62), respectively. The prevalent category of sexual orientation, measured by the Kinsey scale [10], was for trained subjects the category “exclusively heterosexual” (38.7%; 31/80), followed by the category “predominantly heterosexual, only incidentally homosexual” (18.7%; (15/80). Similarly, 45% of untrained subjects (18/40) declared having an exclusively heterosexual orientation, while 22.5% (9/40) reported having a predominantly heterosexual, only incidentally homosexual orientation. Other demographics are reported in Table 1.

### Characteristics Suggestive of Prostitution are Less Prevalent in Trained Sexual Assistants

A significant proportion of untrained subjects (95%; 38/40) reported that their motivation in carrying out sexual assistance was of a remunerative nature, while this proportion was smaller (61.2%; (49/80) among the trained assistants (p = 0.001). While 53.7% (43/80) of trained sexual assistants always received a fee or a gift for their sexual assistance, the percentage of untrained sexual assistants who received a remunerative fee was significantly larger (87.5%; (35/40) (p<0.0001). When participants were asked to specify their activities related to sexual assistance, nearly all untrained subjects (95%; (38/40) claimed to do one or more of the following activities during sexual assistance: sexual intercourse, oral sex, and masturbation. The percentage of participants who engaged in such activities during sexual assistance was much smaller (47.5%; (38/80) in the group of trained subjects, with a significant gap between the trained and untrained assistants (p<0.0001). Thus the percentage of sexual assistance with characteristics of prostitution was 95% (38/40) among untrained and 47.5% (38/80) among trained sexual assistants (p<0.001); Table 2).

**Table 2:** The table shows the questions investigating characteristics suggestive of prostitution.

ITEMS	TRAINED SUBJECTS (N = 80)	UNTRAINED SUBJECTS (N = 40)	P value
**Motivations to work as sexual assistant** * **(%; N)**			
For money	**61.2%; (49/80)**	**95%; (38/40)**	**0.0001**
To turn my sexual interest into a job	**36.2%; (29/80)**	**27.5%; (11/40)**	**0.451**
To take care of people affected by disabilities	**33.7%; (27/80)**	**25%; (10/40)**	**0.442**
I don’t know	**3.7%; (3/80)**	**5%; (2/40)**	**0.872**
**Is your work as a sexual assistant always remunerated by money** **or other kinds of gifts? (%; N)**			
Yes	**53.7%; (43/80)**	**87.5%; (35/40)**	**<0.0001**
No	**46.2%; (37/80)**	**12.5%; (5/40)**	**<0.0001**
I’d rather not say	**0%; (0/80)**	**0%; (0/40)**	**1**
**What kind of activities do you carry out during sexual** **assistance? (%; N)**			
Sexual activities resembling those of prostitution (oral sex, masturbation,sexual intercourse)	**47.5%; (38/80)**	**95%; (38/40)**	**<0.0001**

*Note that some subjects chose more than one option. Hence, the total number of responses does not equal 100% of the sample.

### An Atypical Sexual Interest is more Prevalent among Untrained Sexual Assistants

Sexual fantasies about or overt sexual attraction to disabled people was reported by 42.5% (17/40) of untrained and 12.5% (10/80) of trained sexual assistants (p<0.0001). Twenty per cent of untrained (8/40) and 7.5% (6/80) of trained assistants did not feel distress due to their sexual attraction to people affected by disabilities and satisfied our criteria for an atypical sexual interest [8]. On the contrary, 22.5% (9/40) of untrained and 5% (4/80) of trained assistants (p = 0.009) felt distress due to their sexual attraction to people affected by disabilities. This could suggest that these subjects may be affected by a paraphilic disorder.

We further investigated what the sexual assistants considered most attractive in people affected by disabilities. What appears to be of great interest is that 35% (11/40) of untrained and 3.7% (3/80); (p<0.0001) of trained sexual assistants offered comments that could potentially indicate dangerous behaviours. In particular, these comments may suggest sadistic traits such as the attraction to disabled people’s obedience and impotence, the desire to subjugate disabled people, the possibility of satisfying the assistants’ sexual fantasies, and the attraction to the sexual inexperience that many people affected by disabilities have.

## Discussion

Sexual assistance has aspects that could resemble prostitution and others suggesting that sexual assistants may be similar to subjects with a specific sexual interest in disability (devotees) [8]. This study represents the first attempt to investigate these two relationships in a population of subjects who define themselves as sexual assistants. In particular, we investigated whether rigorous selection and training by specialised organisations may reduce the risk of training subjects with an atypical sexual interest or behaviours resembling prostitution.

The first interesting finding is that the vast majority of trained sexual assistants exhibit behaviours resembling prostitution less frequently than untrained sexual assistants do. Lobbyists and associations dedicated to protecting people with disabilities are strongly committed to the idea that sexual assistance is very different from prostitution, and the professional figures involved in this activity must have a strong sense of altruism and a great open-mindedness. Sexual assistance may be understood as prostitution when the remunerative or commercial character is coupled with specific sexual activities, such as sexual intercourse, oral sex, masturbatory activities, or any activity which involves physical contact and the use of one person by another for her/his sexual satisfaction: in other words, when the exchange becomes “the provision of sexual services for money or its equivalent” [6], [7]. We found that both trained and untrained sexual assistants provide their services under remunerative or commercial conditions. However, the main difference between these two groups lies in the activities carried out. The trained group mainly provides massage therapy, including erotic massage, and discussion about sexuality, contraception, and the appropriate use of sex toys. These activities cannot be strictly considered prostitution. A minority of trained assistants refer to being engaged in masturbatory activities or sexual intercourse that, on the contrary, may be considered activities more typical of prostitution. However, these activities are mainly provided outside of their job as sexual assistants. In contrast, untrained sexual assistants provide sexual services such as oral sex, masturbation, or sexual intercourse much more frequently. These findings seem to confirm that when sexual assistants are trained and carefully selected by specialised organisations, they provide services and activities that do not resemble prostitution. On the contrary, when untrained or opportunistic sexual assistance is provided, it is more likely that these “services” have characteristics similar to prostitution. It should be clarified that when sexual assistance has characteristics similar to prostitution, this is not necessarily negative. In fact, an ethical evaluation of the sexual choices of the sexual assistants is beyond the scope of our study. Furthermore, it is well known that people affected by disabilities find it much more difficult to satisfy their normal sexual interest than other individuals do. For the large majority of people affected by disabilities, sexual assistance represents the unique possibility to explore their sexuality in a safeguarded setting. In this manner the principle that “sexual pleasure is a fundamental right that should be available to all” [11] may also count for people affected by disabilities.

Another interesting finding of our study is the identification of subjects with an atypical sexual interest in disability [9] within the sexual assistant groups. These subjects were characterised by the presence of sexual fantasies about or sexual attraction to disabled people. These behavioural aspects were found especially in the group of untrained sexual assistants, who also report experiencing distress over their sexual interest more often than trained subjects do. This information may suggest that untrained sexual assistants experience more sexual attraction to disability.

The findings of the existence of subjects with an atypical sexual interest among sexual assistants may have significant consequences from a practical point of view. It is well known that people affected by physical, and still more by mental disabilities, are particularly vulnerable to sexual violence [12], [13]. This risk is often due to a lack of employment, strained personal finances, or the desire for a relationship and social connections [13]. However, consideration needs to be given to the potential risk factors of sexual violence that personnel involved in sexual assistance may commit against disabled people. This is highlighted by the answers to the question “What do you find most attractive about people affected by disabilities?” Some attractions reported by the subgroup of assistants with atypical sexual interest may look like expressions of sadistic behaviour, such as the attraction to disabled people’s impotence and obedience or the possibility of satisfying the assistants’ own sexual fantasies. These attractions might give rise, in some circumstances, to the abuse of people affected by mental disabilities.

This study presents some limitations. First, this is an internet-based study, which means that it is based on a unsanctioned methodology for gathering data, implying the impossibility of exerting control over the participants’ environment and of contacting subjects directly. However, in certain research fields, such as sexuality [8], [14], studies refer to some advantages of the internet that make research easier [15]. In particular, the anonymity and the elimination of the uneasiness caused by face-to-face interviews can encourage more honest participation [16], [17]. In addition, the internet may provide a unique opportunity to achieve geographical and cultural diversity in samples [17].

Secondly, the subjects studied here were recruited from online forums and chat groups dedicated to sexual assistance, from associations organising educational training for sexual assistants, and from associations for prostitutes. For this reason, these data may be not representative of the whole population of sexual assistants. However, we did our best to include the largest number of internet data on sexual assistance possible.

Thirdly, we focused especially on female sexual assistants for heterosexual people affected by disabilities. Other subsets composed of male sexual assistants for homosexual males or heterosexuals belonging to different associations of sexual assistants and prostitutes from other countries not considered in this study will have to be explored, in order to make more representative the sample. However, considering that for a number of reasons both physical and intellectual disabilities affect males much more than females [18]–[20], and that, irrespective of the sociocultural aspects, female sex workers are much more common than their male counterparts, we speculate that our experimental setting reflects the largest part of this phenomenon.

Finally, using a non standardised questionnaire might expose us to criticisms, even if this methodology represents the most effective methodology to collect psychometric and demographic data on this new phenomenon.

Future perspectives will require a clear understanding of the phenomenon of sexual assistance and a standardised methodology in order to continue providing people affected by disabilities with the opportunity to explore their sexuality while ensuring their psychological and physical safety.

## Conclusions

Sexual assistance provides a way for people affected by disabilities to attain the right to explore their sexuality in a safeguarded setting. This goal is more effectively achieved if sexual assistants are trained and carefully selected by specialised organisations. It would be of interest to evaluate, as future purpose, the impact the professional sexual assistance might have on the implementation of disabled people’s quality of life.

## Supporting Information

Appendix S1 **Websites dedicated to sexual assistance and prostitution.**
(DOC)
