# Supplementary material for: The Psychosexual Profile of Sexual Assistants: An Internet-Based Explorative Study
Source: PLoS One. 2014 Jun 11;9(6):e98413. doi: 10.1371/journal.pone.0098413 (PMC4053354; doi:10.1371/journal.pone.0098413)
Supplement: Appendix S1 — Websites dedicated to sexual assistance and prostitution. (DOC) [file pone.0098413.s001.doc]

**APPENDIX S1**

www.sindacatosfida.org

www.accaparlante.it

www.fondazioneares.com

http://www.mugsy.org/wendy

http://www.assistancesexuelle.ch

http://www.sexualassistenz.ch

http://www.pianetabile.it/default.asp?id=238

http://www.fabs-online.org/de/home-fabs/fabs

http://www.federsex.com/index.php

http://www.soldiblog.it/post/1027/servizi-di-assistenza-sessuale-per-disabili-l%E2%80%99altra-faccia-della-prostituzione

http://www.isbbtrebel.de/

http://www.isbb-zuerich.ch/

http://www.disabili.com/

http://www.tlc-trust.org.uk/services/index.php

http://www.cosrt.org.uk/index.asp

http://www.madonna-ev.de/

http://www.sexworker.at/

http://www.sexualbegleitung.com/

http://www.koopkoma.de/

http://stjamesinfirmary.org/

http://www.renegadecast.com/

http://test.sfsi.org/

http://subratosensharma.tripod.com

http://swop-nyc.org

http://www.bayswan.org

http://www.iusw.org/

http://www.nswp.org

http://www.scarletalliance.org.au/

http://www.mash.org.uk

http://www.chezstella.org

http://www.spoc.ca

http://www.steppingstonens.ca/

http://www.cabiria.asso.fr

http://www.swop.org.au

http://www.feministe.us

http://www.sexworkersallianceireland.org

http://www.outsiders.org.uk

http://danskhandicapforbund.dk

http://www.iusw.org/

http://www.bestpracticespolicy.org/index.html

http://www.desireealliance.org/

http://www.shada.org.uk/?q=node/2

http://www.sfc.org.uk/

http://www.disabled.gr/lib/

http://sw5.info/

http://www.uknswp.org/index.asp

http://www.touchingbase.org

www.soros.org

http://www.punterlink.com

http://www.saafe.info/

https://fetlife.com

http://www.punternet.com

http://www.puntingzone.com

http://www.sussexscene.com

http://forums.punterplanet.com

http://www.sexyescortads.com

http://tartanladies.com/escort-advertising.html

http://www.scot-pep.org.uk/

http://www.behinderte-leidenschaft.de/leidenschaft/links.html

http://www.fkk-artemis.de
